# Virus infection of phytoplankton increases average molar mass and reduces hygroscopicity of aerosolized organic matter

**DOI:** 10.1038/s41598-023-33818-4

**Published:** 2023-05-05

**Authors:** Ben P. Diaz, Francesca Gallo, Richard H. Moore, Kay D. Bidle

**Affiliations:** 1 Department of Marine and Coastal Sciences, Rutgers University, New Brunswick, USA; 2 NASA Langley Research Center, Hampton, VA USA; 3 NASA Postdoctoral Program, Oak Ridge Associated Universities, Oak Ridge, TN USA

**Keywords:** Virus-host interactions, Microbial ecology, Microbiology, Environmental microbiology, Atmospheric science

## Abstract

Viral infection of phytoplankton is a pervasive mechanism of cell death and bloom termination, which leads to the production of dissolved and colloidal organic matter that can be aerosolized into the atmosphere. Earth-observing satellites can track the growth and death of phytoplankton blooms on weekly time scales but the impact of viral infection on the cloud forming potential of associated aerosols is largely unknown. Here, we determine the influence of viral-derived organic matter, purified viruses, and marine hydrogels on the cloud condensation nuclei activity of their aerosolized solutions, compared to organic exudates from healthy phytoplankton. Dissolved organic material derived from exponentially growing and infected cells of well-characterized eukaryotic phytoplankton host-virus systems, including viruses from diatoms, coccolithophores and chlorophytes, was concentrated, desalted, and nebulized to form aerosol particles composed of primarily of organic matter. Aerosols from infected phytoplankton cultures resulted in an increase in critical activation diameter and average molar mass in three out of five combinations evaluated, along with a decrease in organic kappa (hygroscopicity) compared to healthy cultures and seawater controls. The infected samples also displayed evidence of increased surface tension depression at realistic cloud water vapor supersaturations. Amending the samples with xanthan gum to simulate marine hydrogels increased variability in organic kappa and surface tension in aerosols with high organic to salt ratios. Our findings suggest that the pulses of increased dissolved organic matter associated with viral infection in surface waters may increase the molar mass of dissolved organic compounds relative to surface waters occupied by healthy phytoplankton or low phytoplankton biomass.

## Introduction

Atmospheric aerosols act as cloud condensation nuclei (CCN) affecting cloud radiative forcing and microphysical properties with consequence on hydrological cycles and Earth’s climate. Aerosol chemical composition influences the ability of particles to uptake water and activated to CCN. The aptitude of aerosol particles to form cloud droplets is described by the Köhler theory, which combines the Kelvin effect and Raoult’s Law to predict the equilibrium water vapor saturation ratio around a droplet. The critical size and supersaturation at which an aerosol activates into a water droplet can be represented by a single unitless variable, the hygroscopicity parameter, kappa (κ)^1^ which includes the effects of surface tension and osmolarity^2,3^. In marine regions, where CCN concentrations are low and clouds are particularly sensitive to changes in aerosol properties and CCN concentrations, the understanding of CCN budget and activities is critical to accurately predict weather and climate changes^4^. Microbial communities living near the surface ocean are known to release a complex mixture of organic material less than 0.2 µm in diameter, operationally defined as dissolved organic matter (DOM)^5,6^. DOM can be entrained into aerosols^7^, constituting a significant source of organic aerosol in the marine boundary layer^8^, and can affect the particles’ ability to form CCN^9,10^.

Viral infection of microbes is pervasive in marine environments, with 10^23 ^ infections estimated to happen every second^11,12^. Viruses themselves are colloidal organic particles, ranging in size from 20 to 220 nm in diameter^13^ and are enriched in the sea surface microlayer^14^, as well as within aerosols^15,16^. Virus concentration can vary throughout a phytoplankton bloom in response to changes in mixer layer depth (MLD), with the highest concentration of particles occurring during the bloom decline (~ 10^7^ virus particles ml^−1^), coinciding with phytoplankton death biomarkers^17^. Notably, the range of diameters of marine viral particles^13^ share common CCN activation size diameters and electrical mobility number size distributions in the Northwest Atlantic^18,19^. Evidence from the Northwest Atlantic and sub-Arctic Pacific shows that aerosols are most enriched in organic matter after peaks in phytoplankton concentration^20,21^, suggesting that organic matter derived from phytoplankton demise or viral infection is entrained in aerosols. Indeed, viral lysis of phytoplankton results in the accumulation of viral particles and a mixture of high molecular weight organic material originating from bursting cells^22^, including transparent exopolymer particles (TEP)^23,24^. Phytoplankton viruses have been observed in primary marine aerosols by microscopy^15^ and DNA sequencing^16^, as well as within dust and above the atmospheric boundary layer via flow cytometry^25,26^. Virus infection of phytoplankton alters DOM compared to healthy cells ^27,28^, but the effect of viral infection, including the complex suite of DOM from lysed cells compared to healthy cells and individual virus particles, on CCN activity remains unclear.

Hydrogels are positively buoyant substances^29^ that are a primary constituent of DOM in the ocean and exist over a continuum of sizes, from micrometers to colloidal (~ 0.2 µm to 1 nm diameter) to “truly dissolved” (< 1 nm diameter) precursors^30–33^. TEP particles constitute a portion of marine hydrogels operationally defined by filtration and staging methods^34^. Marine phytoplankton and bacteria produce TEP, in response to different abiotic (e.g., nutrients)^35–37^ and biotic stressors (e.g., virus infection)^38^, which can function as a sticky, adsorptive polymer within the water column and is associated with aerosols with distinct physical properties dependent on local physical conditions^33^. Phytoplankton cells that are stressed and lysing as a result of virus infection release and accumulate hydrogels/TEP, along with a diverse composition of dissolved organic carbon (DOC)^23,38^. As a result, TEP and viruses are enriched in the sea surface microlayer and in aerosols^14,33,39^ compared to the underlying water column. Viruses have also been observed embedded within hydrogels in the water column^40^and within aerosols^41^. Acidic carbohydrates are a primary constituent of hydrogels and are a common moiety found in organic aerosols over ocean regions^42^. Xanthan gum, a chemically defined and commercially available proxy for marine hydrogels and TEP^34^, has also been shown to act as a CCN when mixed with pure water^43^.

Seasonal measurements of surface (down to 5 m depth) TEP during the annual North Atlantic phytoplankton bloom were highest during the late Spring ‘Climax’ phase, when MLDs had recently shallowed and phytoplankton biomass was near its peak concentration for the year^17,44^. Resident phytoplankton were also shown to have high markers of cellular reactive oxygen stress (reactive oxygen species, oxidized lipids), which supports previous studies documenting a physiological link between oxidative stress and TEP production^45^. Phytoplankton accumulation and viral-mediated demise follow predictable seasonal patterns detectable by ocean remote sensing^46–48^, highlighting the potential for incorporating viral infection in marine aerosol cloud models if it resulted in primary aerosols with altered CCN activity^26^.

Taken together, these prior observations suggest that viruses, and the DOM associated with virus infection and bloom decline, are important constituents of sea spray aerosols that could critically impact aerosol properties, atmospheric composition, and cloud formation. Here, we examine how these different types of seasonally varying, ecosystem-linked-DOM, namely virus particles, virus lysates and associated marine hydrogels, affect cloud forming activities. We specifically explored how viral infection and marine hydrogel addition impact the cloud forming activity of aerosols generated from diatoms, coccolithophores, and chlorophytes, all common taxa in the North Atlantic^49,50^, compared to healthy exudates, seawater controls, and purified viruses. Based on the aforementioned studies we hypothesized that viral-induced cell lysis of phytoplankton will decrease the hygroscopicity of DOM compared to healthy phytoplankton DOM along with increasing the molar mass. We also hypothesized that TEP additions will aggregate molecules within aerosols, making them appear to have a higher molar mass due to its sticky nature.

### Methods

#### Growth media and phytoplankton host strains

Phytoplankton hosts were selected to represent those commonly present in North Atlantic seasonal bloom^17,49,50^. Cultures of naked and calcified *Emiliania huxleyi* CCMP374 cells^51^ were grown in f/2-Si amended autoclaved seawater. *Micromonas pusilla* CCMP834 cells were grown in K amended autoclaved seawater, and *Chaetoceros tenuissimus* 2–10 cells were grown in SWM-3 amended autoclaved seawater. Temperature and photoperiod was chosen to be consistent with previously reported culturing conditions for these organisms and represents typical euphotic zone temperatures and light levels in the North Atlantic^52^. *E. huxleyi* and *M. pusilla* were grown in triplicate 3 L acid-washed glass Erlenmeyer flasks at 18 °C on a 16:8 light:dark photoperiod at 150 µmol photons m-^2^ s^-1^ irradiance. *C. tenuissimus* cultures were grown in triplicate 3 L acid-washed glass Erlenmeyer flasks at 15 °C on a 14:10 light: dark photoperiod at similar light intensities. Yuji Tomaru kindly provided *C. tenuissimus* cultures and Anne-Claire Baudoux kindly provided *M. pusilla* CCMP834 as part of a collaborative exchange.

#### Phytoplankton growth and virus infection conditions

Infection experiments used well-characterized coccolithophore, diatom and chlorophyte hosts-virus systems^53–56^. A separate infected culture was grown simultaneously and infected during the exponential growth phase (Supplementary Fig. 1). Naked and calcified *E. huxleyi* cultures were infected with *EhV207 at a* 10:1 virus:host ratio^51^, while *M. pusilla* cultures were infected with *MicV-C* at a 5:1 virus:host ratio. *C. tenuissimus* cultures were infected with 0.5% (v/v) of either *CtenRNAV* or *CtenDNAV* lysate. Uninfected, control phytoplankton cultures were grown to late exponential phase in their respective media. Infected phytoplankton cultures were harvested after they had dropped one to three orders of magnitude from their peak cell concentration (Supplementary Fig. 1). Locally estimated scatterplot smoothing (LOESS) lines for growth curves were drawn using the "geom_smooth” function within the ggplot2 package in R.

#### Processing of phytoplankton lysates and exudates

Phytoplankton control cultures and virus lysates were prefiltered with glass fiber filters (Pall Corporation, A/E then A/C; nominal pore sizes 1.0 µm, 0.7 µm, respectively), concentrated and diafiltered via tangential flow filtration (PALL Corporation Centramate™ LV cassette holder, CM018LV, 10kDA filter, OS010T12) to remove salts. Note that 10 kDa is a nominal size cutoff for this TFF system; a fraction of smaller particles was likely retained regardless of shape (manufacturer’s notes). Purified xanthan gum (XG, Sigma Aldrich G1253) was added as a treatment at levels similar to the highest range of TEP detected in the North Atlantic bloom (200 μg L^−1^)^17^. See Supplementary Fig. 2 for a summary of phytoplankton processing steps and Supplementary Methods for further details.

#### Determination of organic carbon, chloride, sulfate concentrations

Total organic carbon was quantified for desalted, 0.2 µm-filtered, dialyzed samples (3–5 technical replicates) using a Shimadzu TOC-L Analyzer 680C Combustion Catalytic Oxidation system with NDIR Detection Method, equipped with combustion tube for High Salt Samples). Given the solution was filtered with a 0.2 µm pore-size filter prior to analysis, this value is referred to as dissolved organic carbon (DOC) throughout this study. Chloride and sulfate concentrations were also determined for three technical replicates using a Dionex Aquion Ion Chomatraph. Median values from replicates were used for organic carbon, chloride, and sulfate concentrations.

#### CCN measurements

The desalted solutions were nebulized to form aerosol droplets, which were subsequently dried by a silica gel diffusion dryer to produce dry particles composed of a mixture of the organic and inorganic solution components. The particles were then size-classified with a differential mobility analyzer (DMA; TSI 3081A) before being sampled by the CCN Counter (DMT CCN-100)^57^. Scanning Mobility CCN Analysis^58^ was used to determine the aerosol critical activation diameter, as the CCN instrument supersaturation was varied stepwise from 0.27% to 1.71% by increments of 0.24%. The CCN instrument supersaturation was precisely calibrated using ammonium sulfate aerosols, where the supersaturation-dependent hygroscopicity was calculated from Kӧhler Theory^59^, using the ion-interaction approach to compute the osmotic coefficient^60,61^. The DMA sizing was verified using NIST-traceable polystyrene latex spheres, where the plumbing delay time was adjusted slightly to account for the extra tubing needed to split the flow between the CCN counter and a condensation particle counter (CPC, TSI 3776). See Moore et. Al. 2010 for further details and schematic of aerosol sampling setup^58^.

#### Kӧhler theory analysis

The solution chemistry obtained from the chloride, sulfate and DOC measurements were used with Kӧhler’s thermodynamic model to derive the molar mass and surface tension depression of the CCN^3,62^. Since the surface tension depression of a surfactant is known to scale with the concentration of the surfactant, the carbon concentration of the CCN at the point of activation (C_act_) were calculated as follows:

1 $${C}_{act}=\frac{27}{8}{x}_{c, org}{\rho }_{org}{\varepsilon }_{org}\left(\frac{{d}_{c}s}{A\frac{\sigma }{{\sigma }_{w}}}\right)$$

where ${x}_{c, org}$ is the mass fraction of carbon in the organic matter (assumed to be 0.29 following Moore et, al. 2008^3^ and Biddanda 1997^63^),${\rho }_{org}$ the organic dry density (assumed to be 1.4 g cm^−3^),${\varepsilon }_{org}$ is the organic volume fraction, ${d}_{c}$ is the CCN critical activation diameter at the given instrument supersaturation (*s*), $\sigma$ is the surface tension of the CCN at the point of activation, and ${\sigma }_{w}$ is the surface tension of pure water. The parameter A is defined as:

2 $$A=\frac{4{\sigma }_{w}{M}_{w}}{R{T}_{pw}}$$

where R is the gas constant and T is the temperature in Kelvin. The droplet surface tension is assumed to be that of pure water in order to calculate the parameter A. Surface tension depression ($\frac{\sigma }{{\sigma }_{w}})$ is then used in **(1)** and (**3)** to account for the presence of surfactants.

**Figure 1:**
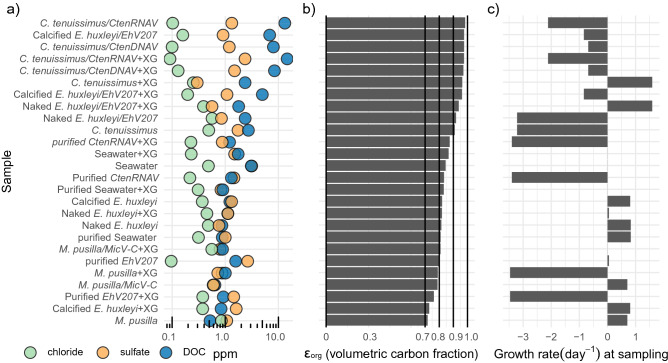
Range of dissolved ions and organic matter associated with healthy exudates, viral lysates and purified virus preparations from various host-virus combinations. (**a**). Concentration of chloride (green), sulfate (orange), and DOC (blue) in parts per million (ppm). (**b**)  ${\varepsilon }_{org}$ (Volumetric carbon fraction) of aerosolized samples. (**c**) Growth rate (d^−1^) of phytoplankton cells at the time of sampling. Negative growth rates refer to net cell death (e.g., during lytic virus infection). Seawater controls correspond to sterilized seawater (and hence growth rate is zero). XG = Xanthan gum. Growth rates were derived from growth curves and are shown in Supplementary Fig. 1. See Table 1 for more detailed descriptions of host and virus characteristics.

The molar mass (M_org_) of aerosolized solutions was calculated as follows:

3 $${M}_{org}=\frac{{P}_{org}{\varepsilon }_{org}{v}_{org}}{\frac{4}{27}{\left(A\frac{\sigma }{{\sigma }_{w}}\right)}^{3}\left(\frac{{p}_{w}}{{M}_{w}}\right){d}_{c}^{-3}{s}^{-2}-\sum_{i\ne org}\left(\frac{{p}_{i}}{{M}_{i}}\right){v}_{i}{\varepsilon }_{i}}$$

where ${v}_{org}$ is the van’t Hoff factors of the organic species (assumed to be unity) and ${v}_{i}$ is the van’t Hoff factors of the inorganic species. The volume fractions of the inorganic species, ${\varepsilon }_{i}$ and the organic species, ${\varepsilon }_{o}$ were calculated from the IC sulfate and chloride anion concentrations and the TOC concentrations, where it was assumed that the inorganic salts were fully neutralized by sodium (See Fig. 1).

To derive the unknown parameters M_org_ and $\sigma$ in **(3)**, we assumed that $\sigma$ =${\sigma }_{w}$ at the lowest CCN supersaturations where the droplet is most dilute. We assumed that M_org_ did not vary with increasing supersaturation (i.e., the composition of the nebulized aerosols is internally mixed and does not vary with size) to calculate at the higher supersaturations, where the droplet is more concentrated (as computed by **(1)**).

In addition to the detailed thermodynamic calculations from Kӧhler Theory, we also computed the particle hygroscopicity (κ) following Petters and Kreidenweis, 2007^1^, and used the IC- and TOC-derived solute volume fractions and theoretical inorganic κ’s to obtain the hygroscopicity of the organic species using a simple, volume-weighted mixing rule:

4 $$\upkappa =\sum_{j}{\varepsilon }_{i}{\upkappa }_{i}$$

**Figure 2:**
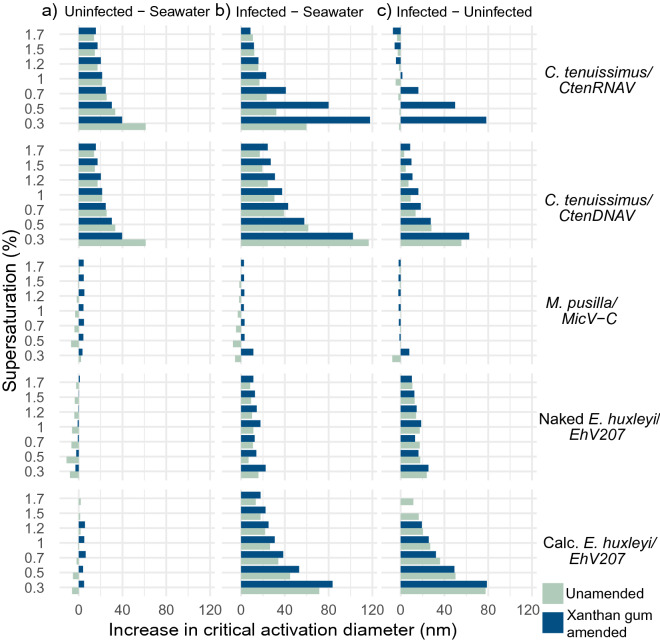
Viral infection increases critical activation diameter across different supersaturations Enrichment of critical activation diameters for (**a**) exudates from uninfected controls and (**b**) infected lysates both compared to the background organic matter in seawater and (**c**) compared to each other, across supersaturations (y axis) and for different host-virus combinations (labeled on right; Calc.=calcified). Bars are colored by the absence (teal) or addition (dark blue) of xanthan gum, a proxy for marine hydrogels (see "Methods"). The same uninfected control is shown for *C. tenuissimus* in panel b), given the same host was used for both CtenDNAV and CtenRNA infections. Un-normalized critical activation diameter curves for this enrichment analysis are found in Supplementary Fig. 2.

This kappa parameter is affected both by solute-dependent changes in the organic molar volume (M_org_ / ${\rho }_{org}$), as well as the surface tension depression ($\sigma /{\sigma }_{w}$). The solute molar volume is not expected to vary with CCN supersaturation, while the surface tension would be expected to change. Consequently, for surface-active solutes, we would expect the critical supersaturation diameter data points to cross lines of constant kappa as supersaturation levels varied (see Fig. 2).

## Results

### Phytoplankton host-virus systems and dissolved organic matter characterization

Five different host-virus combinations were used in this study, along with autoclaved, natural seawater, to elucidate the impacts of different DOM sources on aerosol properties. Host-virus systems spanned diatoms, coccolithophores and chlorophytes, all representative taxa in the North Atlantic, and different virus particle sizes, genome types (dsDNA, ssRNA, ssDNA) and virion compositional properties (enveloped or not) (Table 1). Given the known impact that salts have on aerosol and CCN properties, salts were removed to disentangle the effect of organic matter (See "Methods"). This allowed us to enrich and maximize the organic mass fraction across sample treatments to achieve organic fractions of ~ 70–98% (Fig. 1b).

**Table 1:** Properties of phytoplankton hosts and viruses used in this study.

Host phytoplankton	Virus name	Virus diameter (nm)	Lipid envelope	Genome type	Virus mL^-1^ * lysate/ purified virus
*C. tenuissimus* 2–10	*CtenRNAV*^53^	31	No	ssRNA	2.4*10^9^–2.8*10^9^/ 3*10^7^–1.8*10^7^
*C. tenuissimus* 2–10	*CtenDNAV*^54^	37	No	ssDNA	4.7*10^8^–1.4*10^9^
*M. pusilla* 834	*RCC4229*^55^ (called *MicV-C* in this study)	120	No	dsDNA	4.3*10^7^–3.8*10^7^
*E. huxleyi* 374 (Naked)	*EhV207*^56^	180	Yes	dsDNA	1.4*10^9^–1.3*10^9^/ 2.6*10^8^–1.2*10^8^
*E. huxleyi* 374 (Calcified)	*EhV207*^56^	180	Yes	dsDNA	1.2*10^9^–1.3*10^9^

*Each value before “- “ denotes unamended virus concentration; second value denotes virus concentration for the same lysate amended with xanthan gum. Set of numbers before “/” denotes virus concentration in lysates; numbers after “/” denote purified virus concentrations (see methods for details). Note that only two purified viruses were tested in this study (*CtenRNAV* and *EhV207*).

Virus infection resulted in growth rate reductions from uninfected cultures between 0.65 to 4.03 d^−1^ at time of harvesting and sample processing (Fig. 1c), providing a snapshot of organic matter associated with distinct physiological states. After salt removal and 0.2 µm pore-size filtration, the organic carbon content (hereafter referred to as DOC) of infected lysates, purified viruses and uninfected controls ranged from ~ 0.8 to ~ 13 ppm, while chloride and sulfate ion concentration ranged from ~ 0.1 to ~ 4 ppm (Fig. 1). Virus concentrations in resulting lysates ranged from ~ 10^7^ to ~ 10^9^ virus particles mL ^−1^ (Table 1), with no detectable virus particles in the uninfected controls. Neither bacteria nor phytoplankton cells were detected in any sample. We confirmed that desalted *CtenDNAV*, *CtenRNAV*, and *EhV207* from naked *E. huxleyi* viruses retained their infectivity, killing hosts when incubated at 20:1 virus:host ratio (data not shown). *MicV-C*, and *EhV207* from calcified *E. huxleyi* did not kill their hosts at this ratio.

### Effect of viral infection and hydrogels on critical activation diameter

We compared the CCN critical activation diameter of desalted, unamended seawater with different desalted host and virus treatment combinations to exclude seawater background dissolved organic matter^64^. Overall, higher DOC was found in viral lysates, and was associated with increased the critical activation diameter of aerosols (Supplementary Fig. 3) across host-virus combinations. Aerosolized DOM derived from uninfected and infected phytoplankton cultures shifted the aerosol size distribution of all particles to a larger diameter peak compared to unamended seawater (Supplementary Fig. 4). Viral infection had different impacts on critical activation diameter depending on the specific host and virus combination. The CCN activation diameters of both uninfected and infected *C. tenuissimus* cultures were larger by 5–50 nm compared to that of unamended seawater depending on the supersaturation tested (Fig. 2a, b, Supplementary Fig. 3). On the other hand, uninfected *E. huxleyi* and *M. pusilla* cultures were the same activation diameter as unamended seawater (Fig. 2a). Infection of *C. tenuissimus* with *CtenDNAV* increased the critical activation diameter compared to uninfected *C. tenuissimus* by up to 50 nm (Fig. 2c, Supplementary Fig. 3). However, infection of *C. tenuissimus* with *CtenRNAV* caused no change in critical activation diameter compared to the uninfected exudate. (Fig. 2c, Supplementary Fig. 3). Infection of the naked and calcified phenotypes of the coccolithophore E. huxleyi with *EhV207 s*hifted the critical activation diameter ~ 5–80 nm larger than the critical activation diameter of unamended seawater and exudates from uninfected controls (Fig. 2b, c). In contrast to the other host-virus systems, DOM derived from uninfected *M. pusilla* and from its respective viral lysate did not significantly change CCN activation diameter or aerosol size distribution compared to seawater controls (Fig. 2, Supplementary Fig. 3).

The addition of xanthan gum mostly altered the activation diameter of *CtenRNAV* cultures (Fig. 2; dark blue bars). The largest change resulting from xanthan gum addition was found for *CtenRNAV-*infected cultures at the lower supersaturation levels tested (0.7%,0.5%, 0.26%), causing the critical activation diameter to increase nearly 120 nm compared to seawater (Fig. 2a) and 80 nm more than uninfected *C. tenuissimus* (2c, Supplementary Fig. 3). Adding XG to uninfected *C. tenuissimus* and *CtenDNAV*-infected cultures, on the other hand, resulted in negligible changes to aerosol activation diameters (Fig. 2, Supplementary Fig. 3).

### Aerosol carbon content depressed surface tension

**Figure 3:**
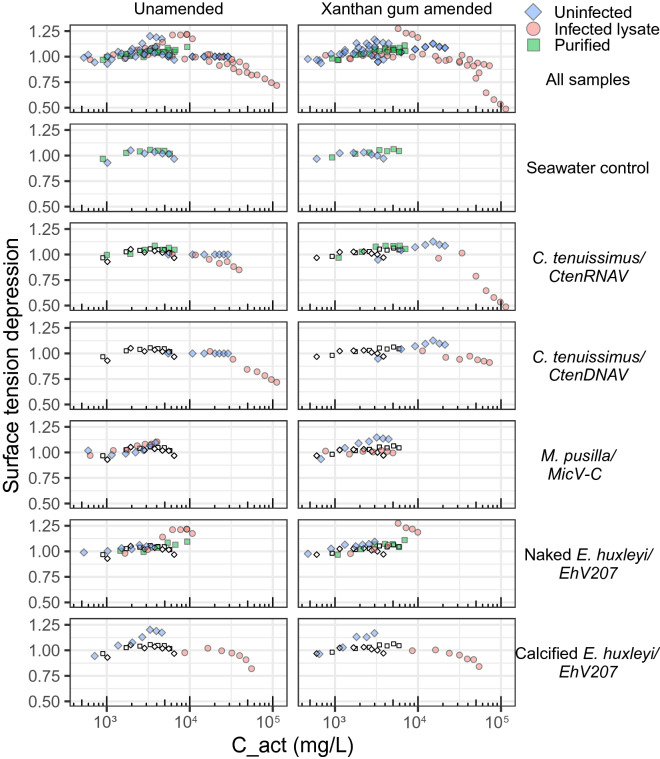
Virus infection increases organic content of aerosol particles and decreases surface tension at sufficiently high organic content. Surface tension depression (from pure water; 1.0 on y-axis, see Methods for calculations) plotted as a function of organic carbon concentration of aerosolized dissolve organic matter. Multiple data points are shown for each sample type (infected lysate, purified viruses and uninfected controls; see color key) to account for the different supersaturations tested (shown in Fig. 2). Open symbols represent unamended seawater control and are plotted for comparison with each phytoplankton culture and virus treatment (labeled on the right).

The increased organic content of aerosol particles in virus infected lysates (over that of uninfected exudates) significant lowered surface tension measured against water ( $\frac{\sigma }{{\sigma }_{w}}$) when organic content exceeded ~ 2*10^4^ mg mL^−1^ (Fig. 3). Purified viruses had similar organic content and surface tension as desalted organic matter from seawater samples, which were themselves similar to pure water (1.0; Fig. 3). Addition of XG further increased the surface tension in *CtenRNAV-*infected cultures of *C. tenuissimus* from 0.5 (unamended) to > 0.9 (XG amended, Fig. 3). XG amendments had the opposite effect on *CtenDNAV* infected cultures of *C. tenuissimus*, decreasing the surface tension from 0.87 (unamended) to < 0.75 (XG amended, Fig. 3). Other than these infected diatom samples, which had high organic content (Table 1), XG addition had negligible effects on surface tension (Fig. 3). Even though calcified *E. huxleyi* lysates also had high organic content (Table 1), addition of XG had little effect on their critical activation diameter or surface tension (Fig. 2, 3), implying that compositional differences in the DOC pools are important factors in aerosol physical properties.

### Impact of viral infection on average molar mass and organic kappa

**Figure 4:**
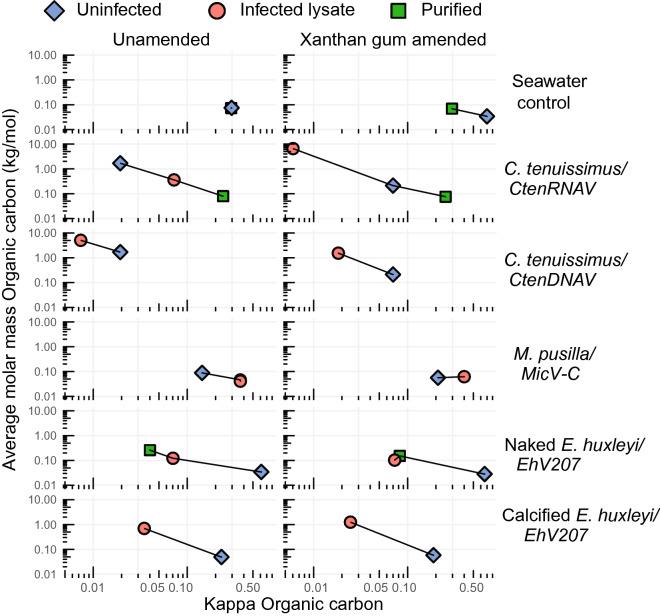
Viral infection decreases kappa and increase average molar mass in three out of five host-virus combinations, while virus infection with xanthan gum amendments decreases kappa and increase average molar mass in four out of five host-virus combinations. Average molar mass of organic carbon (y axis) plotted against organic κ (κ_org;_ x axis) for different lysates of host-virus combinations or organic matter in seawater (control) under unamended (left panels) and xanthan gum amended (right panels) conditions (see Table 1 for more details). Points are shaped and colored by sample type and faceted by whether they were amended with xanthan gum (XG): “Uninfected”, no viruses added (blue diamonds); “Infected lysate”, sampled from an infected culture (pink circles); “Purified”, virus particles purified by ultracentrifugation from infected cultures (green squares). See methods for sample preparation details.

Viral infection lowered the organic κ (κ_org_) compared to seawater and exudates of uninfected hosts across different host-virus combinations (Fig. 4). Aerosols from purified *CtenRNAV* virions had an κ_org_ similar to seawater (κ_org_ ~ 0.3) and higher than its corresponding unpurified lysate (κ_org_ ~ 0.12). Simultaneously, purified *EhV207* virions had a κ_org_ similar to its unpurified lysate (κ_org_ ~ 0.12; Fig. 4), which was also lower than seawater. Despite repeated attempts, we were unable to obtain enough purified and desalted *MicV-C* virions to make similar measurements.

Virus infection increased the average molar mass in three out of five host-virus combinations without XG amendments, and in four out of five host-virus combinations with XG amendments. In both XG amended and unamended treatments, *C. tenuissimus* cultures infected with *CtenDNAV* (5.1 kg mol^−1^ XG unamended) had a higher average molar mass compared to their uninfected control (1.7 kg mol^−1^ XG unamended), and seawater control (0.08 kg mol^−1^ XG unamended). Increases in average molar mass were also observed in the *EhV207-*infected naked (0.12 kg mol^−1^for infected versus 0.03 kg mol^−1^ for uninfected, both XG unamended) and calcified (0.7 infected versus 0.05 kg mol^−1^ uninfected, both XG unamended) *E. huxleyi* cultures (Fig. 4). Notably, infection in *C. tenuissimus* with *CtenRNAV* as well as *M. pusilla* 834 with *MicV-C* neither increased average molar mass nor decreased κ_org_ (Fig. 4). XG additions increased the average molar mass of the *C. tenuissimus* with *RNAV* lysates (0.6 kg mol^−1^ for unamended versus 6.6 kg mol^−1^ for XG amended) and decreased the average molar mass of uninfected *C. tenuissimus* (1.7 kg mol^−1^ unamended versus 0.21 kg mol^−1^ for XG amended) and *C. tenuissimus* infected with *CtenDNAV* (5.1 kg mol^−1^ for unamended versus 1.5 kg mol^−1^ XG amended). XG addition had minimal effect on the other host-virus combinations tested, as well as the organic seawater control.

## Discussion

Here, we characterized the impact of viral infection of eukaryotic phytoplankton on the hygroscopicity of aerosolized dissolved exudates. Our findings highlight a potentially important biological-physical feedback mechanism to phytoplankton bloom demise and cloud formation. The phytoplankton taxa analyzed in this study are widely distributed throughout the world’s surface oceans^49,50,55,65,66^ and their associated viruses have been well-characterized and themselves represent diverse physical and biological characteristics. The viruses analyzed respectively range in size from ~ 30 nm in diameter for the small diatom viruses to ~ 120–180 nm in diameter for representative giant Phycodnaviridae *MicV-C* and *EhV207*, (Table 1) and are coated with a variety of surface proteins and organic matter which likely affect water or salt interactions. For example, *EhV207* virions contain 23 proteins with transmembrane domains^67^, which may be integrated into a lipid envelope^68^. The viruses in this study also contain distinct genome sizes and compositions, comprising dsDNA with hundreds of genes (*EhVs* and *MicV-C*) to ssDNA (*CtenDNAV)* and ssRNA* (CtenRNAV)* with only several genes; hence, they represent different modes and mechanisms of infection altering intracellular organic matter processing and release. Viruses infecting hosts in the same genera as *E. huxleyi, C. tenuissimus* and *M. pusilla* are known to alter the host nutrient storage pathways and intracellular architecture, fundamentally impacting the organic carbon pool during the course of infection and cell lysis^69–71^. The precise differences in the compositional nature of the resulting DOM pools are currently unknown but warrants further investigation.

Our goal was to obtain data from two physiological snapshots from each host-virus combination, namely (1) exudates from healthy, exponentially growing, uninfected control cells, and (2) lysates from late lytic infection, to relate well-characterized physiological states to aerosol properties. The observed results across all cultures tested were generally consistent with the hypothesis that virus infection would lower κ_org_ in aerosols. We observed that aerosolized lysates of some viral-infected cultures had higher inferred average molar mass of aerosol particles (Fig. 4). Previous work showed that viral lysis of cyanobacteria results in release of high molecular weight compounds^72^, which agrees with our own observations of aerosolized viral lysates from eukaryotic phytoplankton (Fig. 4). Further characterization of the organic matter released by host-virus combinations at higher temporal resolution over a range of infected states as cells transition between positive and negative growth rates (as infection takes hold; Supplementary Fig. 1, Supplementary Fig. 5) would provide added insight into the inherent variability of infection on aerosols. Time-resolved processes are likely important to the type of organic matter that can be aerosolized. Indeed, daily sampling of an induced phytoplankton community bloom taken from coastal seawater in Southern California with undefined taxa indicated changes in bacterial biomass and degradation activities throughout the bloom correlated with aliphatic-rich signals in the organic content of sea surface aerosol^73^. This indicates that microbial processing of the organic matter from viral lysates likely alters their respective hygroscopicity^20,21^. Given our samples contained heterotrophic bacteria before removal via filtering, it is worth noting that some of the sampled (and concentrated) DOM had likely undergone enzymatic processing and degradation by bacteria and their corresponding hydrolytic enzymes. Unfortunately, the degree to which this transformation took place is unknown. We argue that this makes our samples relatable to the DOM found in the sea surface, where bacteria are ubiquitous. Given bacteria-mediated alteration of DOM generally scales with the time of exposure to it, it is hard to make direct comparisons between our samples and natural populations, whereby bacterioplankton continuously alter DOM composition until aerosolization.

*MicV-C -*infected *M. pusilla* cells were an exception to the general findings of higher average molar mass induced by viral infection; these aerosols were characterized by slightly lower average molar mass (Fig. 4). The simplest explanation is that our methods were not sensitive enough to detect differences in activation diameter (Fig. 2) or surfactant activity (Fig. 3, 4) because the ratio of organic carbon to salts was too low for this system (Fig. 1a, b). We note that the uninfected *M. pusilla* cultures also had low organic:salt ratios so this appeared to be an inherent property of these taxa (Fig. 1a, b). This could be due to *M. pusilla* losing a higher portion of its dissolved organic carbon during sampling than the other taxa due to the types of filters used (see "Methods"). Alternatively, the inherent differences in host cell physiology such as starch storage compounds found in *M. pusilla*^74^ instead of lipids found in *Chaetoceros* species^75,76^ and *E. huxleyi* ^70,77,78^might affect the cloud forming activity of healthy cell exudates and infected cell lysates.

### Surface tension depression threshold carbon concentration

An important finding in our study was that aerosolized DOM exceeding 2 × 10^4^ mg C L^−1^ depressed surface tension compared to pure water (Fig. 3). Surface tension is an important consideration describing uptake of water by aerosol, as it presents an energetic barrier to expanding into a cloud droplet; lower surface tension will contribute towards a higher hygroscopicity. Moore et, al. 2008 observed decreases of surface tension at more than an order of magnitude lower carbon concentrations^3^ for estuarine DOM at ~ 10^3^ mg C L^−1^, . Key differences between the DOM analyzed in that study may help to explain the disparate thresholds of aerosol carbon concentration required to drop surface tension. Aerosols generated with DOM from *C. tenuissimus* infected with both *CtenRNAV* and *CtenDNAV*, as well as calcified *E. huxleyi* infected with *EhV207,* showed surface tension depressions (Fig. 3), and contained organic carbon content exceeding 97% (Fig. 1b), compared to 85% organic carbon content of surface-active samples found by Moore et al. 2008. Lower ratios of organic carbon to salt have been shown to enhance the surfactant activity of organic matter in aerosol particles due to salting out^2^. Given our sample preparations used a nominal molecular weight cut off of 10 kDa molecular mass (~ 3 nm diameter), it is possible that low molecular mass surfactants^79^ were lost in our sampling process compared to Moore et al. 2008,. Furthermore, Moore et al. 2008 used electrodialysis and reverse osmosis^80^, which retain smaller molecular weight compounds (0.1–1 nm diameter^81^) and may preserve smaller dissolved surface-active compounds released during viral lysis compared to ultrafiltration methods^82^. It’s also possible that estuarine samples were imbued by different inputs of organic matter such as humic acids or cellulose causing increased surface tension depression.

### Xanthan gum amendments has mixed effects

XG was used in this study as a proxy for hydrogels, which are known to exist in a continuum of sizes and alter the physical properties of aerosol^33^. XG addition supplemented a size continuum of DOM ranging from ~ 3 nm to 0.2 μm to for our samples. XG and pure water has been shown to alter aerosol hydration properties^43^, and XG is known to act as an agglomerate coal ash aerosol^83^. We hypothesized that XG might increase the average molar mass of aerosols. Despite its potential binding and hydration properties in aerosols, XG addition only altered aerosol physical properties when added to uninfected and infected *C. tenuissimus.* XG decreased κ_org_ and increased average molar mass when added to *CtenRNAV* infected cultures (as was hypothesized). However, XG increased the κ_org_ and decreased inferred average molar mass of both uninfected and *C. tenuissimus* infected with *CtenDNAV* cultures. Possible mechanisms for these differences could be linked to differential production of TEP and CSP. Both classes of particles are produced during the course of *CtenRNAV* infection^24^, and their ratio varies throughout the course of infection. TEP accumulates throughout infection compared to uninfected cultures, while CSP production is highly variable^24^. Lysates with a higher relative proportion of CSPs would be more impacted by inputs of XG, compared to lysates that already had high concentrations of TEP hydrogels. No large changes in κ_org_ were noted when XG was added to calcified *E. huxleyi* exudates, even at an organic content > 97% (Fig. 1). This implies that high organic content alone does determine whether XG or TEP hydrogels interact to significantly change κ_org_. Further research utilizing metabolomics and higher temporal resolution could determine specific compounds and/or macromolecular classes (e.g., ratio of TEP to CSP) responsible for the differential aerosol dynamics in the presence of XG.

### Low hygroscopicity of diatom cultures

The only organic aerosols to have significantly higher activation diameter, higher inferred molar mass, and lower κ_org_ were those derived from exudates of uninfected *C. tenuissimus* (Fig. 2, 4, Supplementary Fig. 3). These results agree with a previous study which found that diatom DOM was the only phytoplankton exudate type that significantly lowered total κ, even when concentrations of DOC were normalized^9^. The lowered κ_org_ in our study could have been the result of increased organic content as obtained by our concentration methods (Fig. 1b, see "Methods"). Alternatively, the siliceous frustules of *C.* t*enuissimus* (and all diatoms) may have contributed to the low hygroscopicity observed. Silica itself is a poor CCN, so a high abundance of siliceous particles (I.e., intact frustules or pieces of frustules in diatom cultures) may have caused a baseline low hygroscopicity, which was itself altered by the DOM present during both infection and healthy growth. Indeed, mixing pure silica particles with different organic species decreases critical activation diameter to different extents^84^.

### Comparison of CCN activity of purified viruses

Aerosols containing purified haptophyte and diatom viruses were respectively less and equally hygroscopic than aerosols generated from unamended organic matter in seawater (Fig. 4). Aerosols containing pure *EhV207* lowered the organic κ_org_ compared to both unamended seawater organic matter and exdudates from corresponding uninfected host cells (Fig. 4). Purified *CtenRNAV*, on the other hand, had the same κ_org_ value to unamended seawater organic matter and a higher κ_org_ than corresponding exudates from uninfected host culture (Fig. 4). This hints at a fundamental difference in the mechanisms driving lowered κ_org_ in infection lysate of *C. tenuissimus* and *E. huxleyi.* While both infected naked *E. huxleyi* and *C. tenuissimus CtenRNAV* lysates had lower κ_org_ than seawater organic matter (Fig. 4), particles other than viruses appeared to drop the κ_org_ of the latter. As stated above, silica particles have very low cloud forming activity, so they may have driven this trend.

The lowered hygroscopicity of infected *E. huxleyi* lysates instead appears to be attributable to the virus particles themselves. The differences in κ_org_ of purified virus particles from diatoms and coccolithophores may be related to differences in the size and concentration of the virus particles (Table 1), as well as their ability to bind to salt particles during nebulization. *EhV*207 virus particles possess a lipid envelope surrounding the capsid and contain 23 transmembrane proteins which are predicted to be embedded into nonpolar/lipid cell or viral constituents^67^. The hydrophobic lipid envelope may impact *EhV’*s ability to bind with salt during nebulization and lower the κ_org_ of associated aerosols , possibly protecting airborne enveloped viruses and enabling their reported preferred entrainment in aerosols and infectivity after aerosolization^15,16^. Aerosols containing pure *CtenRNAV* may have had insufficient concentration of these small virus particles (~ 30 nm) to detect changes in aerosol properties. There was approximately one order of magnitude less viruses ml^−1^ of purified *CtenRNAV* (~ 3*10^7^) than *EhV207* (~ 3*10^8^), although direct comparisons are not necessarily justified, since two different methods were used for quantification (flow cytometry for *EhV207* and MPN for *CtenRNAV*). Further investigations with similarly sized viruses with or without native lipid envelopes, as well treatments involving lipid envelope removal may confirm if viruses are responsible for lowered hygroscopicity in aerosols. Given viruses infecting *Chaetoceros* might be effective CCN (Fig. 4) and that *Chaetoceros* is one of the most globally abundant genera of diatoms^85^, further investigations into the connection between infection and aerosols are warranted.

### Methodological improvements

The TFF concentration and diafiltration methods used in this study provided novel insights into how the DOM released form healthy and infected cells impact aerosol properties. Improvements in the temporal resolution and organic content yield may help to disentangle some of the observed variability. Our analyses used only a fraction of the total desalted concentrate (~ 7 mL out of ~ 550 mL). Reductions in the initial (phytoplankton culture) and final (TFF concentrate) processing volumes would reduce sample processing effort allowing for increased temporal resolution and yielding over the time course of infections Higher temporal sampling resolution could better disentangle the effects of nutrient limitation-induced senescence and programmed cell death experienced in batch cultures with and without viral infection^45^. Uninfected diatoms and *E. huxeyi* show a range of TEP accumulation throughout growth in culture^35,38,86^, and *M. pusilla, E. huxleyi* and diatoms have distinct TEP and CSP accumulation when infected by viruses^23,24,38^. Furthermore, utilizing a smaller nominal molecular weight cut off (MWCO) in TFF concentration (e.g., 5 kDa; ~ 2 nm)^82,87^ could also theoretically capture a wider range of DOM present in phytoplankton cultures and increase yields, as well as retain more DOM exudates from uninfected cultures for subsequent incorporation into aerosols^22^.

### Atmospheric implications

This study revealed that diverse phytoplankton taxa, and their specific organic matter makeup in response to relevant ecosystem interactions (i.e., virus infection), can impose differences on aerosol properties. These findings could help incorporate phytoplankton bloom dynamics into atmospheric-ocean modeling efforts. Our results indicate that the organic primary aerosol emitted from areas of the ocean with high diatom biomass, regardless of infection state, and in areas of the ocean with declining growth rates of coccolithophores (e.g., due to viral lysis), will have lower hygroscopicity (Fig. 4) and hence make them less able to nucleate clouds. Our results also suggest that pure diatom virus particles may contribute to act as relatively more active CCN. We did not directly measure coccoliths in this study as they are ~1–2 µm in diameter and larger than our size cutoff of 0.2 µm. Coccoliths themselves are preferentially aerosolized in infected cultures versus healthy cultures^88^, and should be considered in modeling cloud formation over infected regions of the ocean, as infection causes coccoliths to be shed from cells^51^.

While we found that the aerosolized organic material associated with some infected phytoplankton and healthy and uninfected diatoms had lower hygroscopicity, the CCN budget would have to be dominated by organics in order to lower the total kappa. In situ studies show that areas of infection have higher dissolved organic carbon entrainment^20,21^ in primary aerosol, which may be a result of organics in these potentially infected regions of the ocean not binding as much salt during bubble bursting/ aerosolization. We measured carbon and salt ion concentrations in the water solutions and not directly on the aerosolized samples in this study so it possible that the aerosolization process itself selectively enriched the organic to salt ratio in some samples, resulting in unique surfactant and CCN activity.

## Conclusion

This study reports the first measurements of how viral infection alters the physical properties of aerosolized organic matter derived from healthy phytoplankton cells and those that had undergone lytic viral infection. Aerosols generated from viral infection lysates with sufficiently high dissolved organic carbon content had an increased critical activation diameter, decreased κ_org_, and detectable surface tension depression compared to unamended seawater and uninfected phytoplankton exudates. The release of dissolved carbon from lytic virus infection also increased the average molar mass of aerosols compared to unamended seawater, contributing to the lower κ_org_ and surface tension depression in some cultures. Aerosols containing pure *EhV207* slightly increased the average molar mass and decreased the κ_org_ compared to unamended seawater, and had no detectable surfactant activity, while aerosols containing pure diatom virus particles did not appear to alter κ_org_, surface tension or average molar mass. Xanthan gum, a proxy for marine hydrogels, altered the surfactant activity of organic matter derived from infected diatom cultures, both increasing (*CtenRNAV*) and decreasing (*CtenDNAV*) the surface tension depression depending on the infecting virus. Our characterization of diverse types of organic matter provides the foundations to link remotely derived phytoplankton growth rates and κ_org_ which are critical to improve the understanding of cloud formation and processes over the open ocean and constrain their representation in climate models.

## Supplementary Information

Supplementary Information.

## Data Availability

The datasets generated during and/or analyzed during the current study are available in the Zenodo repository, https://doi.org/10.5281/zenodo.7490480.

## References

[1] Petters MD, Kreidenweis SM (2007). A single parameter representation of hygroscopic growth and cloud condensation nucleus activity. Atmos. Chem. Phys..

[2] Asa-Awuku A (2007). Alkene ozonolysis SOA inferences of composition and droplet growth kinetics from Kohler theory analysis. Hal. Archives-ouvertes..

[3] Moore RH, Ingall ED, Sorooshian A, Nenes A (2008). Molar mass, surface tension, and droplet growth kinetics of marine organics from measurements of CCN activity. Geophys. Res. Lett..

[4] Moore RH, Karydis VA, Capps SL, Lathem TL, Nenes A (2013). Droplet number uncertainties associated with CCN: An assessment using observations and a global model adjoint. Atmos. Chem. Phys..

[5] Chhun A (2021). Phytoplankton trigger the production of cryptic metabolites in the marine actinobacterium Salinispora tropica. Microb. Biotechnol..

[6] Becker JW (2014). Closely related phytoplankton species produce similar suites of dissolved organic matter. Front. Microbiol..

[7] Miyazaki Y (2018). Chemical transfer of dissolved organic matter from surface seawater to sea spray water-soluble organic aerosol in the marine atmosphere. Sci. Rep..

[8] Spracklen DV, Arnold SR, Sciare J, Carslaw KS, Pio C (2008). Globally significant oceanic source of organic carbon aerosol. Geophys. Res. Lett..

[9] Fuentes E, Coe H, Green D, McFiggans G (2011). On the impacts of phytoplankton-derived organic matter on the properties of the primary marine aerosol – Part 2: Composition, hygroscopicity and cloud condensation activity. Atmos. Chem. Phys..

[10] Cochran RE, Ryder OS, Grassian VH, Prather KA (2017). Sea spray aerosol: The chemical link between the oceans, atmosphere, and climate. Acc. Chem. Res..

[11] Suttle CA (2007). Marine viruses—major players in the global ecosystem. Nat. Rev. Microbiol..

[12] Middelboe M, Brussaard CPDD (2017). Marine viruses: Key players in marine ecosystems. Viruses.

[13] Brum JR, Schenck RO, Sullivan MB (2013). Global morphological analysis of marine viruses shows minimal regional variation and dominance of non-tailed viruses. ISME J..

[14] Aller JY, Kuznetsova MR, Jahns CJ, Kemp PF (2005). The sea surface microlayer as a source of viral and bacterial enrichment in marine aerosols. J. Aerosol Sci..

[15] Sharoni S (2015). Infection of phytoplankton by aerosolized marine viruses. Proc. Natl. Acad. Sci..

[16] Michaud JM (2018). Taxon-specific aerosolization of bacteria and viruses in an experimental ocean-atmosphere mesocosm. Nat. Commun..

[17] Diaz BP (2021). Seasonal mixed layer depth shapes phytoplankton physiology, viral production, and accumulation in the North Atlantic. Nat. Commun..

[18] Kristensen TB (2016). Properties of cloud condensation nuclei (CCN) in the trade wind marine boundary layer of the western North Atlantic. Atmos. Chem. Phys..

[19] Saliba G (2020). Seasonal differences and variability of concentrations, chemical composition, and cloud condensation nuclei of marine aerosol over the north Atlantic. J. Geophys. Res. Atmos..

[20] O’Dowd C (2015). Connecting marine productivity to sea-spray via nanoscale biological processes: Phytoplankton dance or death disco?. Sci. Rep..

[21] Miyazaki Y (2020). New index of organic mass enrichment in sea spray aerosols linked with senescent status in marine phytoplankton. Sci. Rep..

[22] Zheng Q (2021). Highly enriched N-containing organic molecules of Synechococcus lysates and their rapid transformation by heterotrophic bacteria. Limnol. Oceanogr..

[23] Lønborg C, Middelboe M, Brussaard CPD (2013). Viral lysis of Micromonas pusilla: Impacts on dissolved organic matter production and composition. Biogeochemistry.

[24] Yamada Y, Tomaru Y, Fukuda H, Nagata T (2018). Aggregate formation during the viral lysis of a marine diatom. Front. Mar. Sci..

[25] Yahya RZ, Arrieta JM, Cusack M, Duarte CM (2019). Airborne prokaryote and virus abundance over the red sea. Front. Microbiol..

[26] Reche I, D’Orta G, Mladenov N, Winget DM, Suttle CA (2018). Deposition rates of viruses and bacteria above the atmospheric boundary layer. ISME J..

[27] Kuhlisch C (2021). Viral infection of algal blooms leaves a unique metabolic footprint on the dissolved organic matter in the ocean. Sci. Adv..

[28] Xiao X (2021). Viral lysis alters the optical properties and biological availability of dissolved organic matter derived from Prochlorococcus Picocyanobacteria. Appl. Environ. Microbiol..

[29] Azetsu-Scott K, Passow U (2004). Ascending marine particles: Significance of transparent exopolymer particles (TEP) in the upper ocean. Limnol. Oceanogr..

[30] Mari X, Passow U, Migon C, Burd AB, Legendre L (2017). Transparent exopolymer particles: Effects on carbon cycling in the ocean. Prog. Oceanogr..

[31] Azam F, Malfatti F (2007). Microbial structuring of marine ecosystems. Nat. Rev. Microbiol..

[32] Thornton DCOO (2014). Dissolved organic matter (DOM) release by phytoplankton in the contemporary and future ocean. Eur. J. Phycol..

[33] Orellana MV, Hansell DA, Matrai PA, Leck C (2021). Marine polymer-gels’ relevance in the atmosphere as aerosols and CCN. Gels.

[34] Bittar TB, Passow U, Hamaraty L, Bidle KD, Harvey EL (2018). An updated method for the calibration of transparent exopolymer particle measurements. Limnol. Oceanogr. Methods.

[35] Corzo A, Morillo JA, Rodríguez S (2000). Production of transparent exopolymer particles (TEP) in cultures of Chaetoceros calcitrans under nitrogen limitation. Aquat. Microb. Ecol..

[36] Thornton DCO, Chen J (2017). Exopolymer production as a function of cell permeability and death in a diatom (Thalassiosira weissflogii) and a cyanobacterium (Synechococcus elongatus). J. Phycol..

[37] Deng W, Cruz BN, Neuer S (2016). Effects of nutrient limitation on cell growth, TEP production and aggregate formation of marine Synechococcus. Aquat. Microb. Ecol..

[38] Nissimov JI (2018). Dynamics of transparent exopolymer particle production and aggregation during viral infection of the coccolithophore. Emiliania Huxleyi. Environ. Microbiol..

[39] Van Pinxteren M (2022). High number concentrations of transparent exopolymer particles in ambient aerosol particles and cloud water - A case study at the tropical Atlantic Ocean. Atmos. Chem. Phys..

[40] Mari X, Kerros M-EE, Weinbauer MG (2007). Virus attachment to transparent exopolymeric particles along trophic gradients in the southwestern lagoon of New Caledonia. Appl. Environ. Microbiol..

[41] Patterson JP (2016). Sea spray aerosol structure and composition using cryogenic transmission electron microscopy. ACS Cent. Sci..

[42] Hawkins LN, Russell LM (2010). Polysaccharides, proteins, and phytoplankton fragments: Four chemically distinct types of marine primary organic aerosol classified by single particle spectromicroscopy. Adv. Meteorol..

[43] Dawson KW (2016). Hygroscopic growth and cloud droplet activation of xanthan gum as a proxy for marine hydrogels. J. Geophys. Res. Atmos..

[44] Fox J (2020). Phytoplankton growth and productivity in the western north Atlantic: Observations of regional variability from the NAAMES field campaigns. Front. Mar. Sci..

[45] Bidle KD (2015). The molecular ecophysiology of programmed cell death in marine phytoplankton. Ann. Rev. Mar. Sci..

[46] Arteaga LA, Boss E, Behrenfeld MJ, Westberry TK, Sarmiento JL (2020). Seasonal modulation of phytoplankton biomass in the Southern Ocean. Nat. Commun..

[47] Laber CP (2018). Coccolithovirus facilitation of carbon export in the North Atlantic. Nat. Microbiol..

[48] Lehahn Y (2014). Decoupling physical from biological processes to assess the impact of viruses on a mesoscale algal bloom. Curr. Biol..

[49] Bolaños LM (2021). Seasonality of the microbial community composition in the North Atlantic. Front. Mar. Sci..

[50] Kramer SJ, Siegel DA, Graff JR (2020). Phytoplankton community composition determined from co-variability among phytoplankton pigments from the NAAMES field campaign. Front. Mar. Sci..

[51] Johns CT (2019). The mutual interplay between calcification and coccolithovirus infection. Environ. Microbiol..

[52] Śliwińska KK (2023). Sea surface temperature evolution of the North Atlantic Ocean across the Eocene-Oligocene transition. Clim. Past.

[53] Shirai Y (2008). Isolation and characterization of a single-stranded RNA virus infecting the marine planktonic diatom Chaetoceros tenuissimus Meunier. Appl. Environ. Microbiol..

[54] Kimura K, Tomaru Y (2015). Discovery of two novel viruses expands the diversity of single-stranded DNA and single-stranded RNA viruses infecting a cosmopolitan marine diatom. Appl. Environ. Microbiol..

[55] Demory D (2017). Temperature is a key factor in Micromonas-virus interactions. ISME J..

[56] Nissimov JI (2012). Draft genome sequence of the Coccolithovirus Emiliania huxleyi virus 202. J. Virol..

[57] Roberts GC, Nenes A (2005). A continuous-flow streamwise thermal-gradient CCN chamber for atmospheric measurements. Aerosol Sci. Technol..

[58] Moore RH, Nenes A, Medina J (2010). Scanning mobility CCN analysis—a method for fast measurements of size-resolved CCN distributions and activation kinetics. Aerosol Sci. Technol..

[59] Seinfeld JH, Pandis SN (2016). Atmospheric chemistry and physics : From air pollution to climate change.

[60] Pitzer KS, Mayorga G (1973). Thermodynamics of electrolytes. II. Activity and osmotic coefficients for strong electrolytes with one or both ions univalent. J. Phys. Chem..

[61] Clegg SL, Brimblecombe P (1988). Equilibrium partial pressures of strong acids over concentrated saline solutions—I. HNO3. Atmos. Environ..

[62] Padró LT, Asa-Awuku A, Morrison R, Nenes A (2007). Inferring thermodynamic properties from CCN activation experiments: Single-component and binary aerosols. Atmos. Chem. Phys..

[63] Biddanda B, Benner R (1997). Carbon, nitrogen, and carbohydrate fluxes during the production of particulate and dissolved organic matter by marine phytoplankton. Limnol. Oceanogr..

[64] Carlson, C. A., Hansell, D. A. (2015) DOM Sources, sinks, reactivity, and budgets. In *Biogeochemistry of marine dissolved organic matter: 2nd Edn *pp. 65–126 (Elsevier Inc, London) 10.1016/B978-0-12-405940-5.00003-0.

[65] Malviya S (2016). Insights into global diatom distribution and diversity in the world’s ocean. Proc. Natl. Acad. Sci. U. S. A..

[66] Carradec Q (2018). A global ocean atlas of eukaryotic genes. Nat. Commun..

[67] Allen MJ, Howard JA, Lilley KS, Wilson WH (2008). Proteomic analysis of the EhV-86 virion. Proteome Sci..

[68] Mackinder LCM (2009). A unicellular algal virus, Emiliania huxleyi virus 86, exploits an animal-like infection strategy. J. Gen. Virol..

[69] Eissler Y, Wang K, Chen F, Eric Wommack K, Wayne Coats D (2009). Ultrastructural characterization of the lytic cycle of an intranuclear virus infecting the diatom Chaetoceros cf. Wighamii (bacillariophyceae) from Chesapeake Bay, USA1. J. Phycol..

[70] Rosenwasser S (2014). Rewiring host lipid metabolism by large viruses determines the fate of Emiliania huxleyi, a bloom-forming alga in the ocean. Plant Cell.

[71] Bachy C (2018). Transcriptional responses of the marine green alga Micromonas pusilla and an infecting prasinovirus under different phosphate conditions. Environ. Microbiol..

[72] Ma X, Coleman ML, Waldbauer JR (2018). Distinct molecular signatures in dissolved organic matter produced by viral lysis of marine cyanobacteria. Environ. Microbiol..

[73] Wang X (2015). Microbial control of sea spray aerosol composition: A tale of two blooms. ACS Cent. Sci..

[74] Busi MV, Barchiesi J, Martín M, Gomez-Casati DF (2013). Starch metabolism in green algae. Starch - Stärke.

[75] McGinnis KM, Dempster TA, Sommerfeld MR (1997). Characterization of the growth and lipid content of the diatom Chaetoceros muelleri. J. Appl. Phycol..

[76] Buchfink B, Xie C, Huson DH (2014). Fast and sensitive protein alignment using DIAMOND. Nat. Methods.

[77] Vardi A (2009). Viral glycosphingolipids induce lytic infection and cell death in marine phytoplankton. Science.

[78] Malitsky S (2016). Viral infection of the marine alga Emiliania huxleyi triggers lipidome remodeling and induces the production of highly saturated triacylglycerol. New Phytol..

[79] Rosenberg E, Ron EZ (1999). High- and low-molecular-mass microbial surfactants. Appl. Microbiol. Biotechnol..

[80] Vetter TA, Perdue EM, Ingall E, Koprivnjak JF, Pefomm E (2007). Combining reverse osmosis and electrodialysis for more complete recovery of dissolved organic matter from seawater. Sep. Purif. Technol..

[81] Yang Z (2019). a review on reverse osmosis and nanofiltration membranes for water purification. Polymers (Basel).

[82] Erickson HP (2009). Size and shape of protein molecules at the nanometer level determined by sedimentation, gel filtration, and electron microscopy. Biol. Proced. Online.

[83] Zhao Z (2019). Microbial transformation of virus-induced dissolved organic matter from picocyanobacteria: Coupling of bacterial diversity and DOM chemodiversity. ISME J..

[84] Dalirian M (2015). CCN activation of fumed silica aerosols mixed with soluble pollutants. Atmos. Chem. Phys..

[85] Leblanc K (2012). A global diatom database- A bundance, biovolume and biomass in the world ocean. Earth Syst. Sci. Data.

[86] Burns WG, Marchetti A, Ziervogel K (2019). Enhanced formation of transparent exopolymer particles (TEP) under turbulence during phytoplankton growth. J. Plankton Res..

[87] Guo L, Santschi PH (2007). Ultrafiltration and its applications to sampling and characterisation of aquatic colloids. Environ. Colloids Part. Behav. Sep. Characterisation.

[88] Trainic M (2018). Infection dynamics of a bloom-forming alga and its virus determine airborne Coccolith emission from seawater. iScience.

