# Supplementary material for: Virus infection of phytoplankton increases average molar mass and reduces hygroscopicity of aerosolized organic matter
Source: Sci Rep. 2023 May 5;13:7361. doi: 10.1038/s41598-023-33818-4 (PMC10163044; doi:10.1038/s41598-023-33818-4)
Supplement: Supplementary file 1 — Supplementary Information. [file 41598_2023_33818_MOESM1_ESM.pdf]

Supplementary Information

Virus infection of phytoplankton increases average molar mass and reduces hygroscopicity of aerosolized organic matter

Ben P. Diaz[1], Francesca Gallo[2,3], Richard H. Moore[2], Kay D. Bidle[1]

[1] Department of Marine and Coastal Science, Rutgers University, New Brunswick, USA

[2] NASA Langley Research Center, Hampton, Virginia, USA

[3] Oak Ridge Associated Universities, Oak Ridge, Tennessee, USA

Supplementary Methods.
Supplementary Figures 1-3.

Concentration, diafiltration and desalting of phytoplankton cultures. Triplicate 3L cultures were filtered with pre-combusted (550°C, 4 h) glass fiber filters (Pall Corporation, A/E then A/C) to remove most phytoplankton and bacterial cells before concentrating. Glass fiber-filtered cultures were concentrated via tangential flow filtration (TFF) using a 10 kDa pore-size filter (Pall Corporation, 10kDA, OS010T12). Concentration, transferring between glassware, and filtration were performed with Masterflex Pharmapure tubing (L/S 17). The TFF system was cleaned in between samples by running 0.2 µm - filtered MQ water through the system followed by a decontamination solution (20 g sodium hydroxide per L, .025% sodium hypochlorite) for 30 minutes each. The system was soaked overnight in decontamination solution. On the day of sampling, the TFF unit was pre-cleaned by sequentially running fresh, 0.2 filtered MQ water, 1M Sodium acetate buffer, fresh 0.2 filtered MQ water, 0.1M HCl and four changes of fresh filtered MQ water, for 30 minutes each. The lack of residual salts and particles in the tubing lines and filter in the TFF system was confirmed with a conductivity meter (Oakton CON6+, with K=0.1 probe, <0.75 µS/cm). Unamended autoclaved seawater was added to clean lines and filtered for a 5 min pretreatment before starting to concentrate the glass fiber-filtered samples. After the concentrated volume reached 250 mL, unamended autoclaved seawater was added to increase the total volume to 1.25 L. This was done to normalize salt concentrations between different media types and macronutrient levels. This diafiltration was repeated 4 times, after which the sample was concentrated again to ~550 mL total volume. 2 X 100mL of concentrated samples were aliquoted into glass containers for analysis not shown in this study. Salts were removed from the remaining concentrated samples so as not to overwhelm the CCN signal. The remaining ~350mL of concentrate was concentrated down to ~250 mL and filtered MQ water was added to a total volume of 1.25 L. Samples were concentrated to 250 mL. The MQ water dilution and concentration cycle was repeated 8-12 times. The final concentrate was tested for conductivity by aliquoting 4 mL into a 15 mL falcon tube and testing if the conductivity was below 4 µS/cm. 300 mL of the remaining desalted samples were then split into two glass containers. A final concentration of 0.2 mg/L of xanthan gum (Sigma G1253), a typical high value found in the North Atlantic (Diaz 2021) was reached by dissolving xanthan gum in freshly filtered MQ water first (150 rpm, at least 20 min), then adding 660 µL into the 150 mL aliquot and mixing at 150 rpm for 15 min. The xanthan gum and unamended samples were then pumped and filtered into MQ-rinsed precleaned tubing and filtered through a 0.2 µm pore-size filter (Sterivex GV, SVGV010RS), also into pre-combusted glass vials (VWR 71001) for TOC

analysis and polypropylene vials (Nunc, Thermo Scientific 339651) for salt and viral infectivity analyses. Samples were placed in 4°C for 10-30 min before freezing at -20°C and stored for 2-4 months until analyzed.

Cesium chloride purification. Viruses were purified away from the bulk of concentrated dissolved organic matter in concentrated virus lysates (as described above, pre diafiltration) by density gradient ultracentrifugation. 0.2 µm - filtered cesium chloride-amended seawater (1.4 g/mL) was used to create six density gradients, ranging from unamended concentrate to 1.4g/mL. These gradients were gently layered into 12 mL centrifuge tubes (Beckman Coulter Ultra-Clear, 344059) and ultracentrifuged for 4 h at 25,000 RPM at 15°C (r_{av}= 77,100 g, SW 41-T Rotor, Beckman 80-T Ultracentrifuge). A band was extracted via a sterile serological pipette (VWR 89130-869) and stored in a TOC-free glass vial overnight at 4°C. The viral concentrate was diafiltered the following day as described above by first setting up a TFF unit and adding seawater to reach a total volume of 1.25 L. A control sample with only concentrated autoclaved seawater was subject to the same cesium chloride density purification as described above. A similar volume of seawater from this control sample with a similar buoyant density to the viruses was sequentially diafiltered against seawater and filtered MQ water.

Virus enumeration. *EhV207* and Micromonas *MicV-C* were enumerated via flow cytometry as follows. Lysates and uninfected cultures were fixed with a final concentration of 0.5% glutaraldehyde at 4°C for 10 minutes, snap frozen in liquid nitrogen, and stored at −20 °C. Frozen fixed samples were thawed, diluted 1:50 into Tris-EDTA (TE) buffer (pH7.4) and 1X SYBR Green and heated for 10 minutes at 80°C (Brussard 2004). After cooling in the dark, samples were counted on the BD Biosciences Influx Mariner using 488 nm excitation and 520 nm emission. SYBR Green-stained, TE Buffer only controls were used to determine background noise. Sub-micron size calibration beads (Spherotech, 0.2, 0.5 µm diameter) and *EhV207* and *MicV-C* lysates were used to calibrate virus sizes via forward scatter (FSC). *CtenDNAV* and *CtenRNAV* were counted via most probable number (MPN) as in Kranzler et al 2019[1]. Cells from each well were counted after 2 weeks of incubation on a BD Biosciences Accuri flow cytometer. Wells were considered lysed if they fell below a threshold of $5x10^5$ cells mL^{-1}. EPA's MPN tool was used for most probable number estimation. (https://mostprobablenumbercalculator.epa.gov/mpnForm).

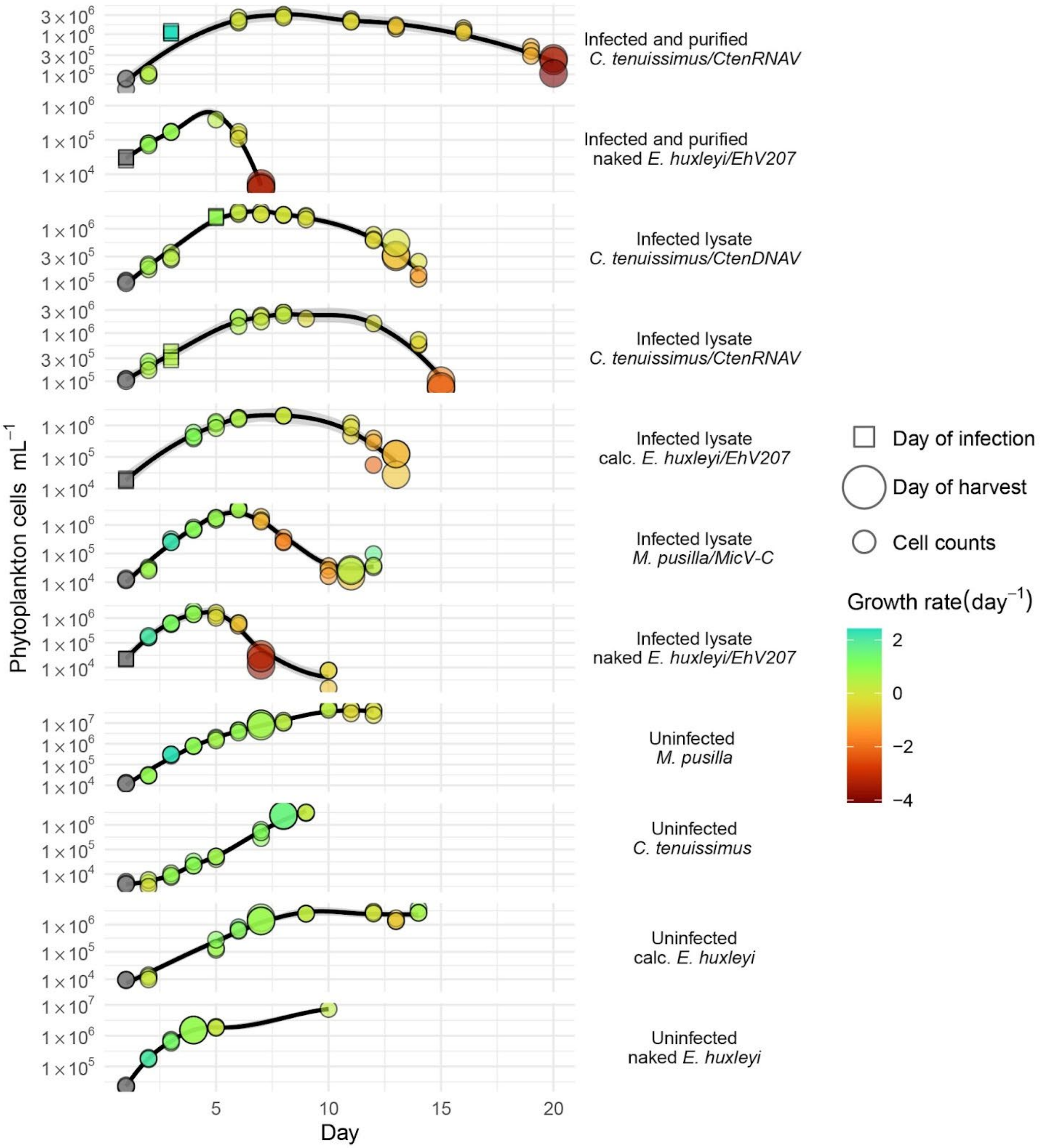

Supplementary Figure 1. Growth curves of phytoplankton culture treatments used in this study. Each panel represents triplicate 3 L cultures of phytoplankton grown with or without virus infection (see labels on right side). Time courses represent sampling over three months; the same time scale (in days) is presented on x-axis for ease of comparison among cultures. Data points are shaped by infection (squares) denoting the day or by "cell counts" (circles). Data points are colored by the growth rate (μ; d-1) at time of sampling (considering the previous day cell counts). Growth rates reported in this study (Fig. 1) represent the median value of triplicates taken on "Day of harvest." In some instances, 30 mL of each culture was

transferred to a 40 mL plastic flask and incubated under the same conditions to confirm that cultures were not infected and present a more complete growth curve.   Larger circles   denote harvest days. The first data point in each growth curve is colored gray to denote that no growth rate numbers are available. Lines drawn between points are LOESS regression, and the shaded gray area represents the 95% confidence interval.

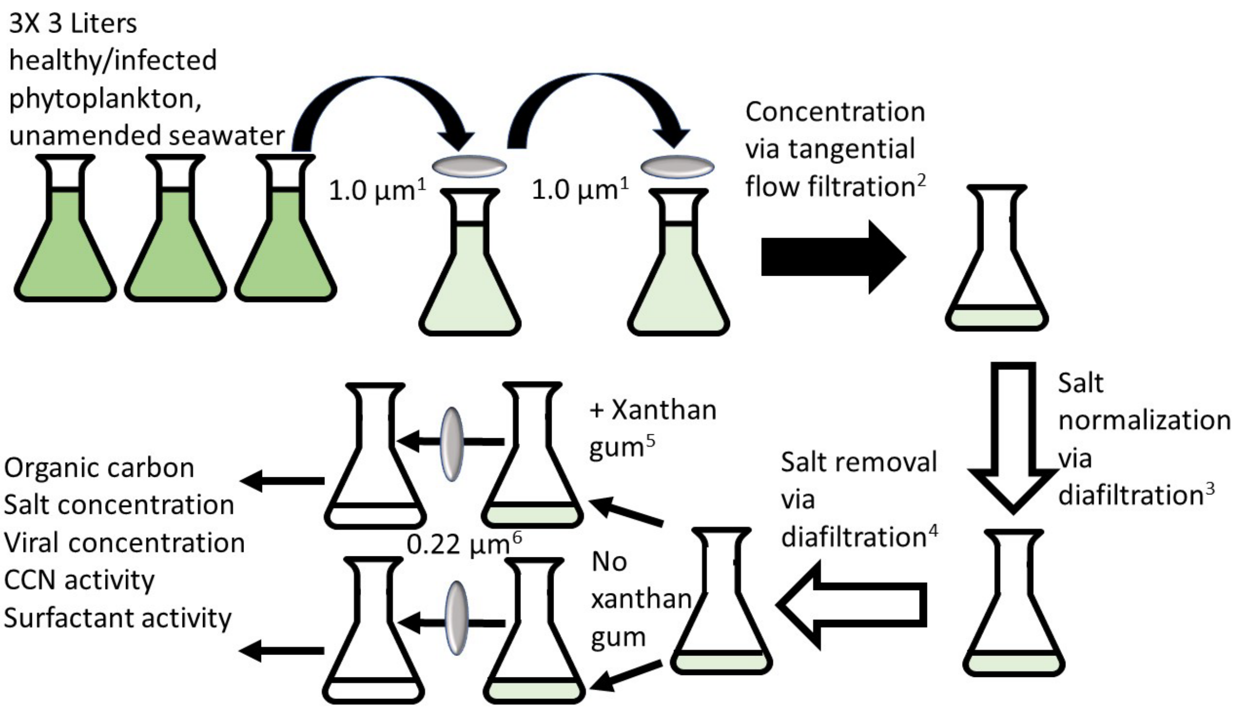

Supplementary Figure 2: Dissolved Organic Material Processing in this Study. Outline of Processing from 9 liters of phytoplankton liquid cultures (healthy, infected and unamended seawater control) to desalted dissolved organic material. **1)** Nominal pore size of glass fiber filters (A/E then A/C, Pall Corporation) used with Erlenmeyer filter flask. **2)** Tangential flow filtration concentrated particles 10 kDa and larger, reducing volume from 9L to ~0.3L (PALL Corporation Centramate™ LV cassette holder, CM018LV, 10kDA filter, OS010T12). **3)** 4 1L flushes of autoclaved unamended seawater (used as base for phytoplankton growth media) was used to normalize salt concentration and dissolved material under 10 kDa. **4)** Fresh MilliQ water was used to flush until conductivity was below 0.8 µS/cm, between 8-14 1L flushes. **5)** Xantham gum in MilliQ water solution was added to a final concentration of 200 µg L^{-1} (Sigma Aldrich G1253), spun at 300 rpm for 15 minutes. **6)** Final filtration through 0.22 micron filter ((Millipore SVGP01015) into glass (VWR 71001) or rinsed polycarbonate centrifuge tubes (VWR 89039) and stored at -20C until further sampling. For details of further sampling see methods and Supplementary Materials.

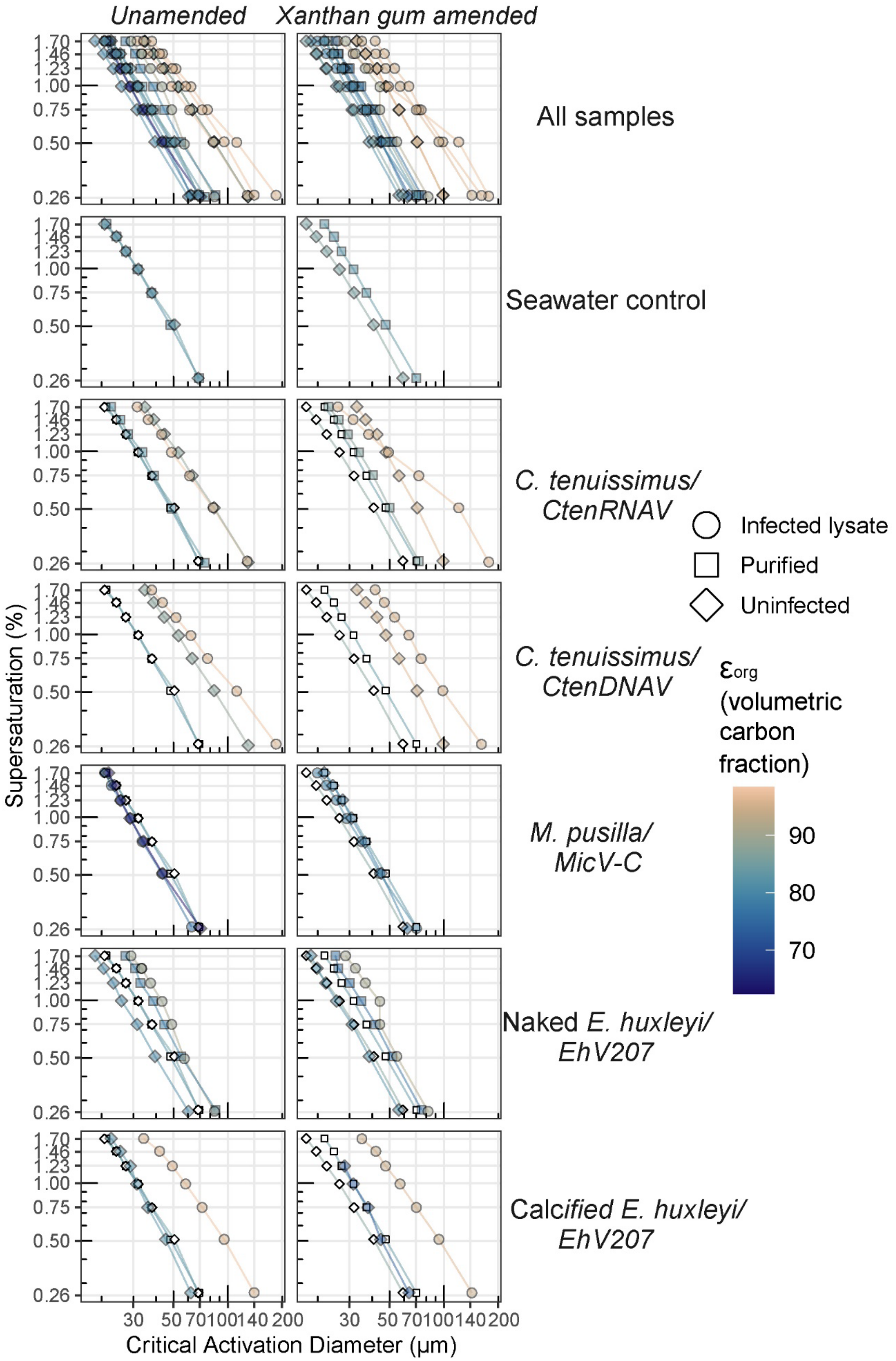

Unamended
Xanthan gum amended
All samples
Seawater control
C. tenuissimus/ CtenRNAV
C. tenuissimus/ CtenDNAV
M. pusilla/ MicV-C
Naked E. huxleyi/ EhV207
Calcified E. huxleyi/ EhV207
Infected lysate
Purified
Uninfected
ε_{org} (volumetric carbon fraction)
90
80
70
Supersaturation (%)
1.70
1.46
1.23
1.00
0.75
0.50
0.26
Critical Activation Diameter (µm)
30
50
70
100
140
200

Supplementary Figure 3. Impact of DOM source on the critical activation diameter of aerosols. Critical activation diameter (x-axis, log_{10}) plotted for different supersaturations (y-axis) of freshly emitted aerosol particles. Samples are grouped by different host-virus combinations (see labels on right); data points are shaped by type of DOM from phytoplankton cultures (infected lysate, uninfected controls, and purified samples; see symbol legend)) and seawater controls (see Methods for details of sample preparation). Data points and lines connecting each sample are colored by $\boldsymbol{\varepsilon_{org}}$ (Volumetric carbon concentration) present in aerosols. Open data points in each panel are seawater controls. Note that virus infection either increases or has no effect on the critical activation diameter of aerosolized phytoplankton dissolved organic matter.

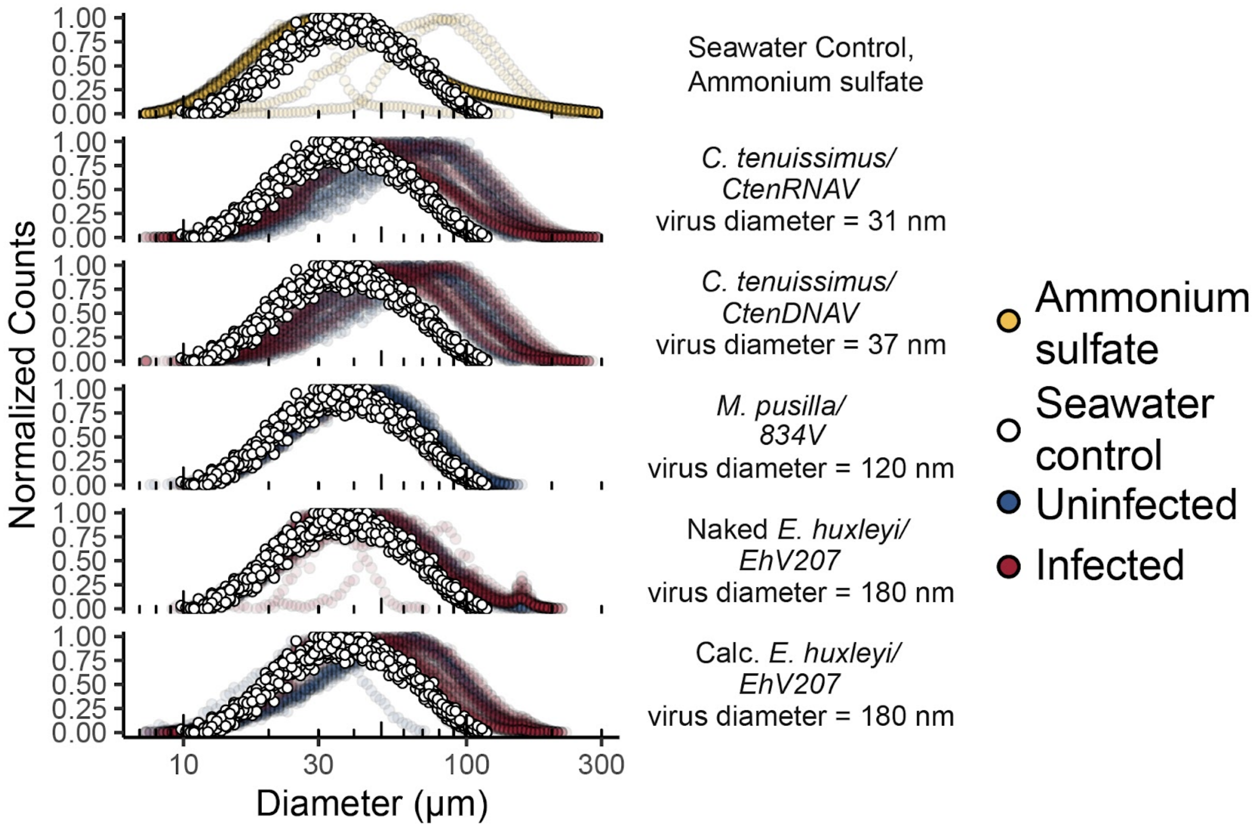

Supplementary Figure 4. DOM derived from healthy and infected phytoplankton cells produces aerosol size distribution with a larger diameter peak than unamended seawater. Counts were taken from the first ten scans of each aerosol sample used in this study and normalized by scan. Points are colored by seawater control (open), ammonium sulfate control (tan), uninfected phytoplankton culture (blue) and infected phytoplankton culture (dark red). Note that both naked and calcified infected *E. huxleyi* cultures have a peak around the same diameter as a virus particle.

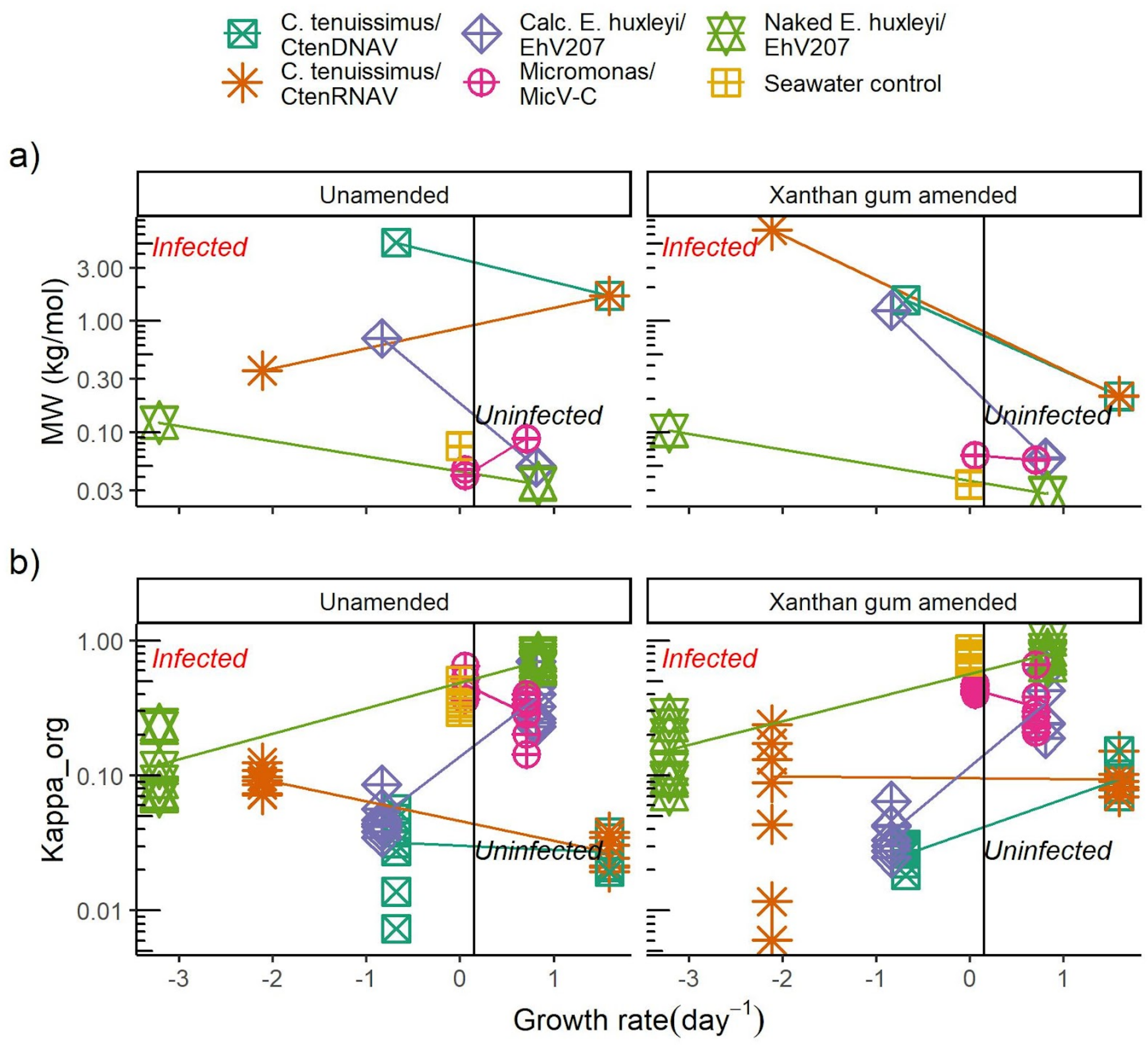

Supplementary Figure 5. Dissolved organic material derived from virus infection and uninfected diatoms increases the average molar mass and decreases kappa of organic material compared to unamended seawater. Impact of growth rate (d^{-1}) on a) average molar mass of aerosolized organic matter and b) Kappa of organic matter (κ_{org}). Plotted growth rates on x-axis refer to respective healthy (black text) and infected lysates (red text) on the day of harvesting samples. Points are shaped and colored by host and virus combinations, with lines connecting mean values of uninfected (right) and infected (left) points for respective taxa (see Table 1 for more sample details). Average molar mass and κ_{org} of purified viruses shown in Supplementary Fig. 4.

REFERENCE CITED

1. Kranzler, C. F. *et al.* Silicon limitation facilitates virus infection and mortality of marine diatoms. *Nat. Microbiol.* **4**, 1790–1797 (2019).
